# Quantitative Analysis and Diagnostic Significance of Methylated SLC19A3 DNA in the Plasma of Breast and Gastric Cancer Patients

**DOI:** 10.1371/journal.pone.0022233

**Published:** 2011-07-18

**Authors:** Enders K. O. Ng, Candy P. H. Leung, Vivian Y. Shin, Chris L. P. Wong, Edmond S. K. Ma, Hong Chuan Jin, Kent-Man Chu, Ava Kwong

**Affiliations:** 1 Department of Surgery, The University of Hong Kong, Hong Kong, Special Administrative Region, People's Republic of China; 2 Department of Molecular Pathology and Department of Surgery, Hong Kong Sanatorium and Hospital, Hong Kong, Special Administrative Region, People's Republic of China; 3 Biomedical Research Center, Sir Run Run Shaw Hospital, Medical School of Zhejiang University, Hangzhou, China; 4 Hong Kong Hereditary Breast Cancer Family Registry, Hong Kong, Special Administrative Region, People's Republic of China; 5 Department of Surgery, Stanford University School of Medicine, Stanford, California, United States of America; University of Barcelona, Spain

## Abstract

**Background:**

Previously, we have examined the methylation status of SLC19A3 (solute carrier family 19, member 3) promoter and found that SLC19A3 was epigenetically down-regulated in gastric cancer. Here, we aim to develop a new biomarker for cancer diagnosis using methylated SLC19A3 DNA in plasma.

**Methodology/Principal Findings:**

SLC19A3 gene expression was examined by RT-qPCR. Methylation status of SLC19A3 promoter was evaluated by methylation-specific qPCR. SLC19A3 expression was significantly down-regulated in 80% (12/15) of breast tumors (P<0.005). Breast tumors had significant increase in methylation percentage when compared to adjacent non-tumor tissues (P<0.005). A robust and simple methylation-sensitive restriction enzyme digestion and real-time quantitative PCR (MSRED-qPCR) was developed to quantify SLC19A3 DNA methylation in plasma. We validated this biomarker in an independent validation cohort of 165 case-control plasma including 60 breast cancer, 45 gastric cancer patients and 60 healthy subjects. Plasma SLC19A3 methylated DNA level was effective in differentiating both breast and gastric cancer from healthy subjects. We further validated this biomarker in another independent blinded cohort of 78 plasma including 38 breast cancer, 20 gastric cancer patients and 20 healthy subjects. The positive predictive values for breast and gastric cancer were 90% and 85%, respectively. The negative predictive value of this biomarker was 85%. Elevated level in plasma has been detected not only in advanced stages but also early stages of tumors. The positive predictive value for ductal carcinoma in situ (DCIS) cases was 100%.

**Conclusions:**

These results suggested that aberrant SLC19A3 promoter hypermethylation in plasma may be a novel biomarker for breast and gastric cancer diagnosis.

## Introduction

Epigenetic changes, such as DNA methylation, are one of the most common molecular alterations in human cancer [1], including gastric [2] and breast cancer [3]. Emerging studies have shown that tumor-specific epigenetic alterations of DNA can be detected from plasma or serum of patients with various malignancies [4], [5], [6]. It has been reported that breast cancer patients contained approximately 4 times more cell-free DNA in serum compared with that of healthy individuals [7]. Recent study has also demonstrated that hypermethylation of RASSF1A DNA in serum is detected in gastric cancer [8] and is associated with a worse outcome in breast cancer patients [9]. The promising results of these studies predict that such circulating markers are likely to be identified with improved diagnostic sensitivity and specificity for cancer. In particular, SLC19A3 (solute carrier family 19, member 3 or THTR-2, thiamine transporter-2) which has been found to be down-regulated in gastric and breast cancer [10], [11], [12], could be a potential candidate for this diagnostic purpose. SLC19A3 as well as SLC19A2 (solute carrier family 19, member 2 or THTR1, thiamine transporter-1) are the carriers responsible for the transportation of thiamine across the plasma membrane [13], [14], [15]. Like folate and many other water-soluble micronutrients, thiamine was transported across the plasma membrane via a specialized carrier-mediated mechanism. It has been recognized for a long time that thiamine deficiency frequently occurs in cancer patients [16]. Previously, we showed that SLC19A3 but not SLC19A2 was frequently down-regulated through promoter hypermethylation in gastric cancer [10]. In the present study, we hypothesized that hypermethylated promoter region of SLC19A3 may serve as a marker for cancer detection. Thus, we developed a simple methylation-sensitive restriction enzyme digestion and real-time quantitative PCR (MSRED-qPCR) assay to quantify SLC19A3 DNA promoter methylation in plasma. The objective was to apply such SLC19A3 MSRED-qPCR to investigate the possibility of using such marker as a novel diagnostic marker for breast and gastric cancer.

## Materials and Methods

### Ethics statement

The study was performed in accordance to the Declaration of Helsinki. Written informed consent was obtained from all participants involved in this study. This study was approved by the Institutional Review Board of The University of Hong Kong/ Hospital Authority West Cluster, Hong Kong.

### Study design and patients

A total of 259 participants were recruited in this study from December 2008 through September 2010. Primary breast cancer and their paired non-tumor breast tissues were collected from 15 breast cancer patients. Plasma samples from 98 breast cancer, 65 gastric cancer and 80 healthy subjects were collected for biomarker validation. Biomarker validation consisted of two phases: (i) Phase I validation: an independent validation cohort of 165 case-control plasma samples including 60 breast cancer, 45 gastric cancer patients and 60 healthy subjects; (ii) Phase II validation: another independent blinded validation cohort of 78 plasma samples including 38 breast cancer, 20 gastric cancer patients and 20 healthy subjects. All samples were collected from patients who underwent surgical resection of tumors. Tissue samples were collected, stabilized with Tissue Stabilization solution (Applied Biosystems), and stored at −80°C. All tumors had been histologically confirmed. Tumors were staged according to the tumor-node-metastasis (TNM) staging system. Informed consent was obtained from all participants. This project was approved by the Institutional Review Board of The University of Hong Kong/ Hospital Authority West Cluster, Hong Kong.

### RNA Extraction and Real-time qRT-PCR

Total RNA was extracted from tissue using Trizol reagent (Invitrogen) according to the manufacturer's instructions. 0.5 µg of total RNA was reversely transcribed into cDNA with High Capacity cDNA Reverse Transcription kit (Applied Biosystems). Real-time qPCR was performed using QuantiTect SYBR Green PCR Kit (Qiagen) in ABI PRISM 7900 HT system (Applied Biosystems). As previously described [10], primers used for SLC19A3 gene expression were: Forward, 5′-TTCTCCATGATGAGACCCTC and Reverse, 5′-ATGATGACTGGCTTGTAGCG. The expression levels of SLC19A3 mRNA were normalized to glyceraldehyde-3-phosphate dehydrogenase (GAPDH). Fold change of SLC19A3 expression was calculated by the equation 2^−ΔΔCt^. ΔCt was calculated by subtracting the Ct values of GAPDH from the Ct values of SLC19A3. ΔΔCt was then calculated by subtracting ΔCt of the control from ΔCt of breast cancer. Real-time qPCR was performed in triplicate.

### Bisulfite Treatment of DNA and Methylation-Specific qPCR

Tissue DNAs were extracted using QIAmp DNA Mini Kit (Qiagen) following the manufacturer's instructions. Genomic DNA (500 ng) extract from tissue was bisulfite-treated with EpiTect Bisulfite Kit (Qiagen) according to the protocol provided. Methylation-specific qPCR was carried out with 2 µl of bisulfite-treated DNA using QuantiTect SYBR Green PCR Kit (Qiagen) in ABI PRISM 7900 HT system (Applied Biosystems) according to the manufacturer's instructions. As previously described [10], methylation-specific primers were: SLC19A3-mF, 5′-TTGGATTTATTCGATAGTCGC and SLC19A3-mR: 5′-CCAACACGCGAACTACGACG, and unmethylation specific primers were: SLC19A3-uF, 5′-TGTTTGGATTTATTTGATAGTTGT and SLC19A3-uR, 5′-CCCAACACACA AACTACAACA. qPCR was performed using QuantiTect SYBR Green PCR Kit (Qiagen) in ABI PRISM 7900 HT system (Applied Biosystems). Percentage of methylation in tissue samples was calculated by the following equation: % meth = 100/[1+2^ΔCt(meth-unmeth)^]% [17]. ΔCt_(meth-unmeth)_ was calculated by subtracting the Ct values of methylated SLC19A3 signal from the Ct values of umnethylated SLC19A3 signal. Each sample is run in duplicates for analysis.

### Plasma DNA extraction and MSRED-qPCR

Plasma from blood samples was processed and stored as described previously [18]. Plasma DNAs were extracted using QIAmp DNA Blood Mini kit (Qiagen), as previously described [19], [20] with modifications. In brief, 600 µl of plasma was used and DNA was eluted with 40 µl of DNase-free water. 30 µl of extracted plasma DNA was digested in a 40 µl reaction volume with 30 U of methylation-sensitive restriction enzyme, BstU1 (New England BioLabs), at 60°C for 16 hours. To ensure complete enzyme digestion, it was run in parallel with a positive and a negative control digestion in which 30 ng of completely methylated or unmethylated control DNA (EpiTect Control DNA, Qiagen) was digested. After digestion, same amount of digested or undigested plasma DNA along with control digestion was subjected to qPCR using QuantiTect SYBR Green PCR Kit (Qiagen) in ABI 7900 HT system (Applied Biosystems). Each reaction is performed in a final volume of 20 µl containing digested (1.3 µl) or undigested (1 µl) plasma DNA, 500 nM each primer and 1× SYBR Green PCR Master mix (Qiagen). At the end of the PCR cycles, melting curve analyses are performed to validate the specific PCR product. Primers used for this qPCR were: Forward, 5′-CCGCGTGCTGGGATTC and Reverse, 5′-TCCAGAAGGCTGCAAATGG. Relative expression level of plasma methylated SLC19A3 DNA was expressed as 2^ΔCt(undigest-digest)^. ΔCt_(undigest-digest)_ was calculated by subtracting the Ct values of digested plasma DNA from the Ct values of undigested plasma DNA. Since Ct of undigest should be ≤Ct of digest, the expression level ranges from 1 to 0. Each sample is run in duplicates for analysis. For 100% digestion efficiency, relative expression level of completely unmethylated control DNA (2^ΔCt(CTRLundig-CTRLdig)^) must be close to zero whereas the level of completely methylated control must be 1.

### Statistical analysis

The significance of plasma level of methylated DNA was determined by Mann-Whitney test. The significance of SLC19A3 gene expression between tumor tissues and their paired adjacent non-tumor was determined by Wilcoxon test. Correlation analysis between SLC19A3 gene expression and methylation percentage was determined by Spearman rank. The ROC value, sensitivity and specificity were calculated according to the standard formulas. All *P*-values are two-sided and <0.05 was considered statistically significant by GraphPad PRISM 4 software (GraphPad Software, La Jolla, CA).

## Results

### Patient characteristics

Patient characteristics and distribution of tumor stages were summarized in Table 1. There were no significant differences of age between breast cancer patients (mean, 58 years, SD = 7.3), gastric cancer patients (mean, 62 years, SD = 8.9), healthy controls (mean, 57 years, SD = 6.5).

**Table 1:** Patient characteristics.

	Breast cancer (n = 98)	Gastric cancer (n = 65)	Healthy Control (n = 80)
Characteristic	No. of patients	No. of patients	No. of patients
Age (years)			
Mean	58	62	57
Median (range)	61 (37–80)	60 (31–80)	59 (39–65)
Gender			
Male	0	32	34
Female	98	33	46
TNM stage			
*DCIS	27	-	-
I	30	12	-
II	27	7	-
III	13	19	-
IV	1	27	-

*DCIS, Ductal carcinoma in situ.

### SLC19A3 is frequently down-regulated through promoter hypermethylation in breast cancer

**Figure 1:**
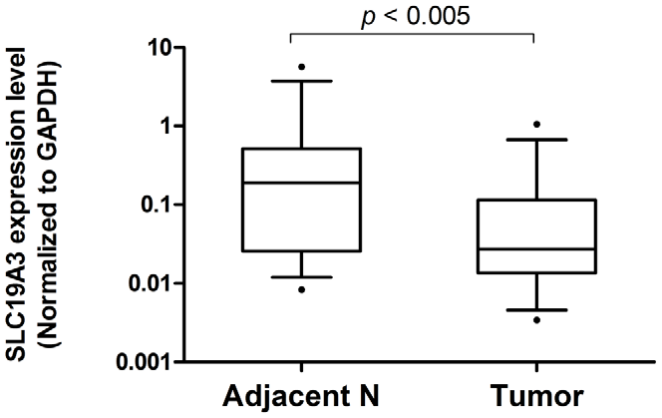
Down-regulated SLC19A3 gene expression in primary breast cancer tissues. Relative SLC19A3 mRNA expression between tumor tissues and their paired adjacent non-tumor breast from breast cancer patients (n = 15) by real-time qPCR. Expression of SLC19A3 mRNA (Log_10_ scale at Y-axis) was normalized to GAPDH. The lines inside the boxes denote the medians. The boxes mark the interval between the 25^th^ and 75^th^ percentiles. The whiskers denote the interval between the 10^th^ and 90^th^ percentiles. Statistical difference was analyzed by Wilcoxon test, *P*<0.005.

**Figure 2:**
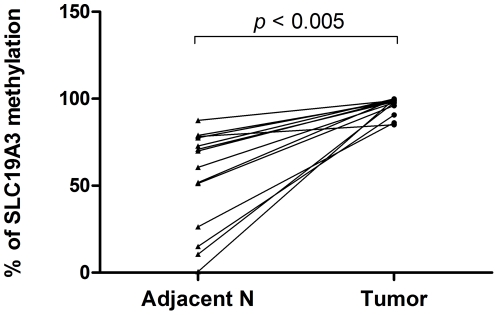
Increased percentage of SLC19A3 DNA methylation in primary breast cancer tissues. Percentage of SLC19A3 promoter methylation between tumor tissues and their paired adjacent non-tumor breast tissues from the 15 breast cancer patients by MS-qPCR. Percentage of methylation in tissue samples was calculated by the following equation: % meth = 100/[1+2^ΔCt(meth-unmeth)^]%. ΔCt_(meth-unmeth)_ was calculated by subtracting the Ct values of methylated SLC19A3 signal from the Ct values of umnethylated SLC19A3 signal. Statistical difference was analyzed by Wilcoxon test, *P*<0.005.

**Figure 3:**
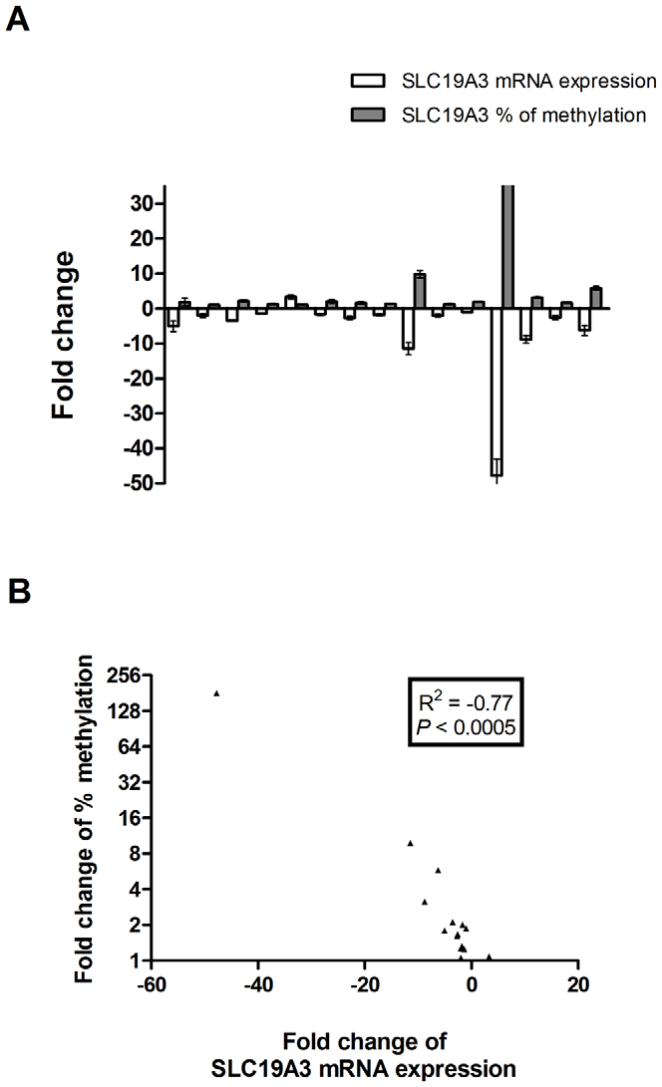
SLC19A3 is frequently down-regulated through promoter hypermethylation in breast cancer. (A) Fold change relationship between SLC19A3 mRNA expression and methylation percentage in those 15 breast cancer patients (mean ± SD). (B) Correlation analysis of the fold changes between SLC19A3 mRNA expression and methylation percentage (Spearman rank correlation, R^2^ = −0.77, *P*<0.0005).

To examine the expression level of SLC19A3 gene, we performed qRT-PCR on 15 pairs of primary breast cancer tumor tissues and their adjacent non-tumor breast tissues from 15 Chinese female breast cancer patients. Our results showed that SLC19A3 mRNA level was significantly decreased in 12 of 15 (80%) breast cancer tumor tissues when compared to their adjacent non-tumor breast tissues (fold change ranging from −2 to −50, *P*<0.005; Wilcoxon paired test; Figure 1). To investigate whether down-regulation of SLC19A3 expression is associated with hypermethylation of SLC19A3 promoter CpG islands, we performed methylation-specific qPCR on those 15 pairs of primary breast cancer tumor tissues and their adjacent non-tumor breast tissues using primers targeting on a hypermethylated promoter CpG region as previously studied [10]. Our data revealed that those breast tumors had significant increase in methylation percentage when compared to adjacent non-tumor breast tissues (*P*<0.005; Wilcoxon paired test; Figure 2). Spearman rank correlation showed that fold changes between SLC19A3 mRNA expression and methylation percentage were negatively correlated (R^2^ = −0.77, *P*<0.0005; Figure 3).

### Phase I validation: Quantitative analysis of methylated SLC19A3 DNA in the plasma of breast and gastric cancer patients

**Figure 4:**
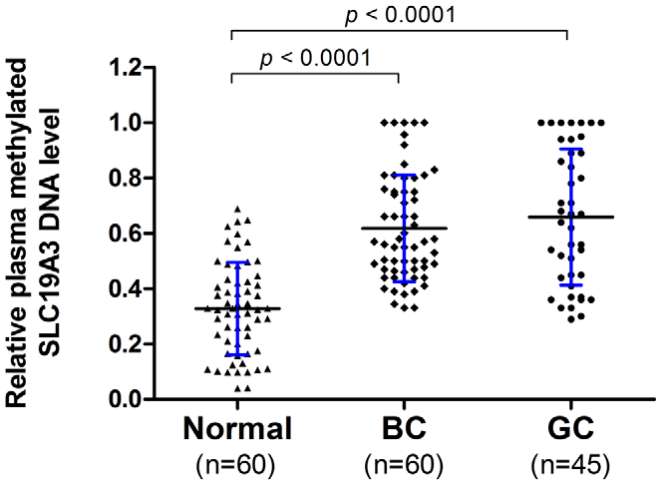
Quantitative analysis of plasma methylated SLC19A3 DNA on a group (n = 165) of plasma samples by MSRED-qPCR. Scatter plots of plasma levels of methylated SLC19A3 DNA in 60 healthy normal subjects, 45 gastric cancer (GC) and 60 breast cancer (BC) patients. Plasma level of methylated SLC19A3 DNA is expressed as 2^ΔCt(undigest-digest)^. ΔCt_(undigest-digest)_ is calculated by subtracting the Ct values of digested plasma DNA from the Ct values of undigested plasma DNA. Since Ct of undigest should be ≤Ct of digest, the expression level is ranging from 1 to 0. The horizontal black lines denote the means. The blue errors bars denote the ± standard deviations (SD). Statistically significant differences were determined using Mann-Whitney *U* tests, *P*<0.0001.

Plasma level of methylated SLC19A3 DNA was assessed on a group of 165 plasma samples including 60 breast cancer patients, 45 gastric cancer patients and 60 healthy controls. Our results showed that methylated SLC19A3 DNA in plasma was significantly higher in both gastric and breast cancer patients than those of controls (All *P*-values<0.0001; Mann-Whitney U test; Figure 4). To examine whether those markers are gender specific, plasma levels of methylated SLC19A3 DNA were compared between the female and male healthy in both gastric cancer group and control group. Our data indicated similar plasma levels of this marker (*P* = 0.344 for gastric cancer, *P* = 0.467 for controls; Mann-Whitney U test) between female and male, suggesting their expressions are not gender specific.

### Phase II validation: Blinded analysis of breast and gastric cancer samples

**Figure 5:**
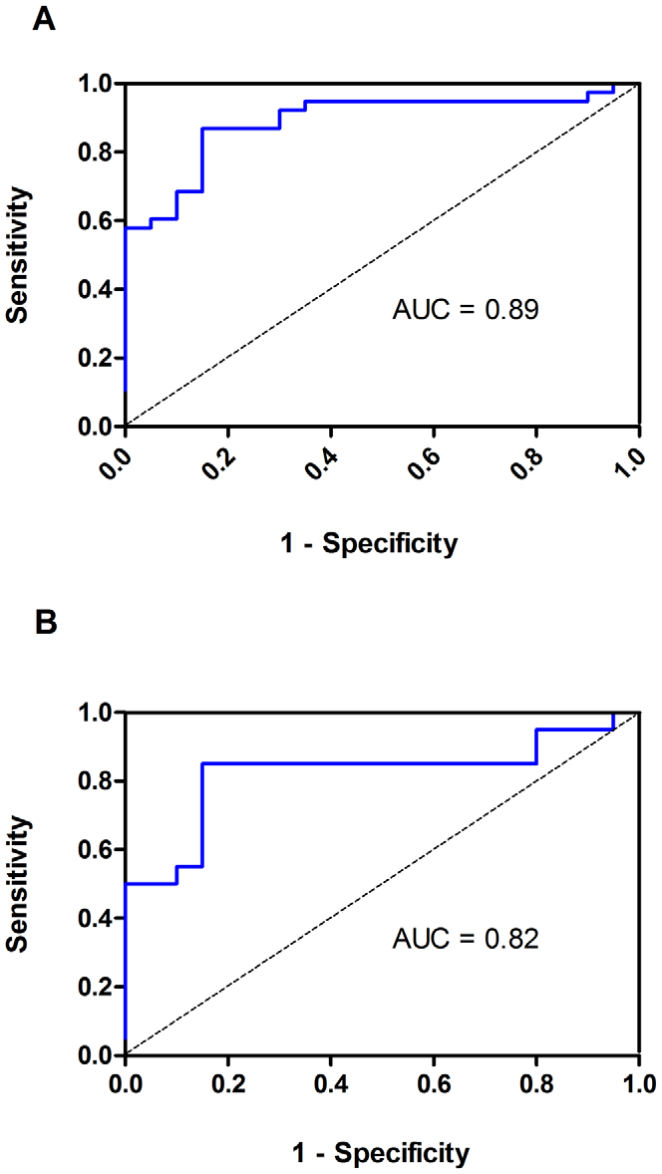
The receiver operating characteristics (ROC) curve for plasma methylated SLC19A3 DNA in the Phase II blinded validation (n = 78). (A) Area under curve (AUC) value was 89%. At cutoff value of 0.41, sensitivity was 87% and specificity was 85% in discriminating 39 breast cancer patients from 20 control subjects. (B) Area under curve (AUC) value was 82%. At cutoff value of 0.41, sensitivity was 85% and specificity was 85% in discriminating 20 gastric cancer patients from 20 control subjects.

**Figure 6:**
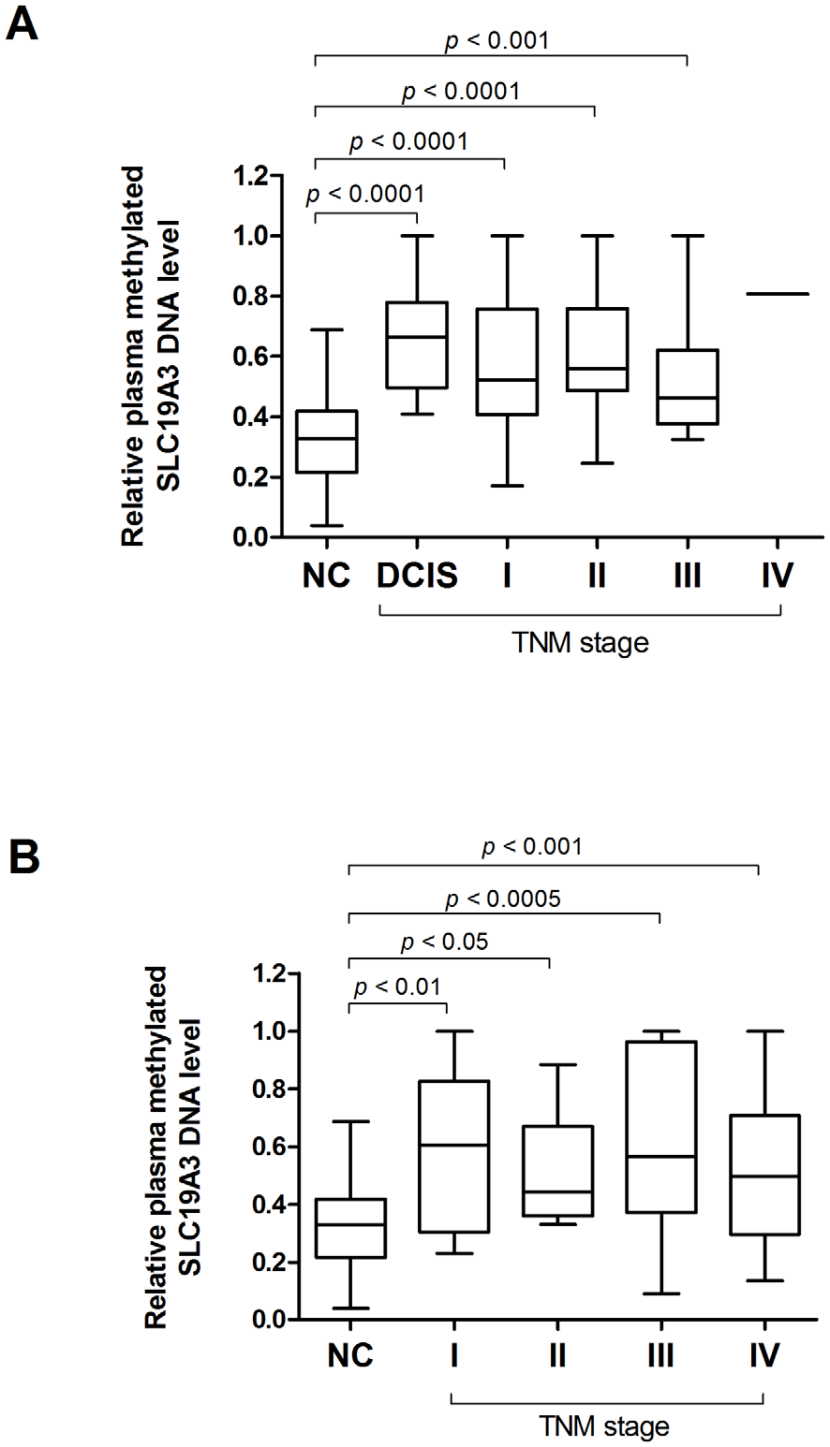
Plasma methylated SLC19A3 DNA levels across tumor stages from both Phase I and II validation. (A) Box plot of plasma methylated SLC19A3 DNA level in 80 NC, 27 ductal carcinoma in situ (DCIS) plus 71 BC patients across various TNM stages. (B) Box plot of plasma methylated SLC19A3 DNA level in 80 NC and 65 GC patients across various TNM stages. The box represents the interquartile range and the line across the box indicates the median value. Relative plasma level of methylated SLC19A3 DNA is expressed as 2^−ΔCt(Dig-Undig)^. ΔCt_(Dig-Undig)_ is calculated by subtracting the Ct values of digested plasma DNA from the Ct values of undigested plasma DNA. Statistically significant differences were determined using Mann-Whitney tests.

In this blinded validation phase, plasma level of this biomarker was assessed on a blinded cohort of 78 plasma samples including 38 breast cancer patients, 20 gastric cancer patients and 20 healthy controls. Our results showed that the positive predictive values for breast and gastric cancer were 90% and 85%, respectively. The negative predictive value of this marker was 85%. For breast cancer, this marker yielded ROC value of 89% (Std. error = 0.044, 95% CI = 80.3% to 97.6%). At a cutoff point of 0.41, sensitivity was 87% (95% CI = 71.9% to 95.6%) and specificity was 85% (95% CI = 62.1% to 96.8%). For gastric cancer, this marker yielded a ROC value of 82% (Std. Error = 0.073, 95% CI = 68.0% to 96.5%). At a cutoff point of 0.41, sensitivity was 85% (95% CI = 62.1% to 96.8%) and specificity was 85% (95% CI = 62.1% to 96.8%) (Figure 5A and 5B). To examine whether the plasma levels of methylated SLC19A3 DNA may be associated with stages of the disease, patients from both Phase I and II validations were stratified based on the diagnosis of TNM staging. Of all gastric and breast cancer patients, plasma levels did not vary significantly across stage of the patients (*P* = 0.149 for breast cancer, *P* = 0.826 for gastric cancer; Kruskal-Wallis test). However, significant differences were obtained when individual tumor stage including early stage was compared with the controls, (All *P*-values<0.05; Mann-Whitney U test; Figure 6A and 6B). Intriguingly, of all DCIS cases (n = 27) from breast cancer patients this plasma marker generated positive predictive value of 100% at the cutoff of 0.41.

## Discussion

In our previous study of gastric cancer, we showed that SLC19A3 but not SLC19A2 was down-regulated through promoter hypermethylation [10]. In this study, we further confirmed that frequent down-regulation of SLC19A3 expression in breast cancer is associated with hypermethylation of its promoter CpG region. To explore the possibility of using this methylated SLC19A3 DNA as a marker for breast cancer detection, we developed a robust and simple methylation-sensitive restriction enzyme-based quantitative assay to measure methylated SLC19A3 DNA in plasma. Our results showed that plasma level of methylated SLC19A3 DNA was significantly elevated in breast cancer patients compared with those of controls. In the blinded validation, our results showed that the positive predictive values for breast and gastric cancer were 90% and 85%, respectively. The negative predictive value of this marker was 85%. At a cutoff point of 0.41 (relative plasma level of methylated SLC19A3 DNA), the sensitivity was 87% and the specificity was 85% in discriminating breast cancer from control subjects. At a cutoff point of 0.41, the sensitivity was 85% and the specificity was 85% in discriminating gastric cancer from control subjects. Intriguingly, at a cutoff of 0.41, the positive predictive value of this biomarker was 100%, which are reasonably good to discriminate early stage DCIS cases from healthy controls. Although this finding suggests plasma level of methylated SLC19A3 may serve as an early diagnostic marker for breast cancer, further validations in larger DCIS cohorts are necessary.

It has been recognized for a long time that thiamine deficiency frequently occurs in cancer patients [16]. Notably, recent study showed that SLC19A3 but not SLC19A2 deficiency in mice leads to an impairment in intestinal thiamin uptake and a decrease in blood thiamin levels, suggesting an important role of SLC19A3 in thiamine regulation [21]. SLC19A3 down-regulation frequently occurred in cancers including breast cancer [11], [12]. Thus, SLC19A3 might function as a novel candidate tumor suppressor gene in cancers. Previously, we are the first to report SLC19A3 is epigenetically silenced in human cancers [10]. Here we demonstrate the potential usefulness of methylated SLC19A3 DNA in plasma for cancer detection. Importantly, elevation of plasma methylated SLC19A3 DNA level has been detected in not only advanced stages but also pre-cancerous DCIS and early stages of tumor suggesting a potential marker for early breast and gastric cancer detection. These results show that hypermethylation of SLC19A3 promoter occurred in the earliest form of cancer.

Instead of using bisulfite modification, we applied MSRED-qPCR. This technology replaces the tedious and low-throughput bisulfite-based methods with a simple restriction enzyme digest and qPCR. DNA methylation-sensitive restriction enzyme is used to selectively digest unmethylated DNA. The remaining DNA after digestion or without digestion is quantified by qPCR using primers flanking the hypermethylated SLC19A3 promoter CpG region. Using this methodology, we can quantify any particular CpG island by simply designing a pair of primers flanking particular region of interest. Unlike bisulfite modification, MSRED is a simple and specific enzyme digestion and subsequent qPCR is performed without DNA purification. This makes methylated DNA in plasma easily detectable. Furthermore, the same primer pair is used to amplify the digests and undigests allowing the PCR results to be reliably and directly compared. In contrast, bisulfite modification-based PCR methods use two different pairs of primers to amplify either methylated or unmethylated templates of a given target sequence. Differences in their amplification efficiencies could cause biased results, and thus requires normalization to methylated reference DNA. Historically, the digestion method has been problematic due to incomplete digestion causing falsely high methylation results. In this regards, incomplete digestion could be eliminated because our protocol used an excess of restriction enzyme and a sufficient incubation time relative to the recommended DNA amounts. Furthermore, control DNA digestion was included to ensure the completeness of digestion in the samples.

Although our results are promising, the sample size is still small. Thus, further validations in large cohorts are necessary. The reliability, simplicity and quantitative nature of this assay make this technology an ideal tool for biomarker development in human cancers. In the future, the combination of other potential markers may further improve the discriminating power of this test.
